# Primary chronic cold agglutinin disease: An update on pathogenesis, clinical features and therapy

**DOI:** 10.1080/10245330701445392

**Published:** 2007-07-21

**Authors:** Sigbjørn Berentsen, Klaus Beiske, Geir E. Tjønnfjord

**Affiliations:** 1 Department of Medicine, Haugesund Hospital, Haugesund, Norway; 2 Department of Pathology, Rikshospitalet-Radiumhospitalet Medical Center and Faculty Division Rikshospitalet, University of Oslo, Oslo, Norway; 3 Department of Medicine, Rikshospitalet-Radiumhospitalet Medical Center and Faculty Division Rikshospitalet, University of Oslo, Oslo, Norway

**Keywords:** B-lymphocytes, cold agglutinin disease, fludarabine, hemolytic anemia, lymphoproliferative, rituximab

## Abstract

Chronic cold agglutinin disease (CAD) is a subgroup of autoimmune hemolytic anemia. Primary CAD has traditionally been defined by the absence of any underlying or associated disease. The results of therapy with corticosteroids, alkylating agents and interferon-a have been poor. Cold reactive immunoglobulins against erythrocyte surface antigens are essential to pathogenesis of CAD. These cold agglutinins are monoclonal, usually IgMκ auto antibodies with heavy chain variable regions encoded by the V_H_4-34 gene segment. By flowcytometric and immunohistochemical assessments, a monoclonal CD20^+^κ^+^B-lymphocyte population has been demonstrated in the bone marrow of 90% of the patients, and lymphoplasmacytic lymphoma is a frequent finding. Novel attempts at treatment for primary CAD have mostly been directed against the clonal B-lymphocytes. Phase 2 studies have shown that therapy with the chimeric anti-CD20 antibody rituximab produced partial response rates of more than 50% and occasional complete responses. Median response duration, however, was only 11 months. In this review, we discuss the clinical and pathogenetic features of primary CAD, emphasizing the more recent data on its close association with clonal lymphoproliferative bone marrow disorders and implications for therapy. We also review the management and outline some perspectives on new therapy modalities.

## Introduction

Autoimmune hemolytic anemia (AIHA) is classified into warm and cold reactive antibody types. Several entities are recognized within the cold antibody group; chronic cold agglutinin disease (CAD), acute cold antibody mediated AIHA complicating *Mycoplasma pneumoniae* or viral infections, and paroxysmal cold hemoglobinuria. Only CAD will be further addressed in this review. CAD has traditionally been classified into a primary or idiopathic type which has been regarded unrelated to underlying conditions, and a secondary type associated with malignant disease, most often lymphoma [1–3]. The term “cold” is primarily derived from the immune biology of CAD, not from the clinical features which will be discussed in detail below [4,5].

Cold hemagglutination was first reported by Land-steiner in 1903 [6] and found to occur in human beings in 1918 [7]. The association of cold hemagglutination with hemolysis was described in 1937 by Rosenthal and Corten [8]. During the 1960s, Dacie [9] and Schubothe [10] published systematic descriptions of 16 CAD patients each. The auto antibodies responsible for hemagglutination at low temperatures, cold agglutinins (CA), may be found in the sera of healthy subjects as well as in patients with AIHA of the cold reactive types [5,9]. CA bind to erythrocyte surface antigens at a temperature optimum of 0–4°C [4,11]. In contrast to polyclonal CA in healthy individuals, monoclonal CA often have a high-thermal amplitude, which contributes to their pathogenicity at temperatures approaching 37°C [4,11–13].

Binding of CA causes agglutination of erythrocytes [9,10,14] and the antigen–antibody complex induces complement (C) activation and hemolysis [15,16]. Essential clinical manifestations of primary CAD are hemolytic anemia and cold-induced circulatory symptoms [9,10,17]. Exact estimates of the severity of anemia and the frequency of cold-induced symptoms, however, have not been provided until recent years [3,9,10,18].

Management was largely unsatisfactory until the last decade [3,19,20]. Recently, considerable progress has been made in the knowledge of clinical features, bone marrow pathology, humoral and cellular immunology, candidate targets for therapy, and more efficient management. We will review relevant findings by our group and others on clinical, immunological and pathogenetic features of primary CAD. Based on these results, we will provide an overview of more recent therapeutic measures and give some suggestions for further studies.

## Epidemiologic and clinical features

In single-center series, primary CAD has been found to account for 13–15% of the cases of AIHA [1,21,22]. In a population-based clinical study of primary CAD in Norway, the prevalence was found to be 16 per million in habitants and the incidence rate 1 per million inhabitants per year [3]. Little is known about possible geographic variations. Median age of CAD patients was 76 years and median age at onset of symptoms was approximately 67 years [3]. The male/female ratio has been reported to be 0.5–0.6 which is not very different from a male/female ratio of 0.72 in an age-matched general population. The frequency of auto-immune disorders other than CAD does probably not differ from what is to be expected in an elderly population with some female predominance [3,4]. Median survival was about 12.5 years from diagnosis and median age at death was 82 years, which implies a life expectancy in these patients similar to that of an age-matched general population [3].

Cold-induced circulatory symptoms, although often not emphasized by physicians, are considered typical for CAD [10,17]. We found that more than 90% of patients with primary CAD had such symptoms, ranging from moderate acrocyanosis to severe Raynaud phenomena precipitated even by very slight cold exposure [3]. Although the importance of cold exposure for exacerbation of hemolysis has been questioned [18], characteristic seasonal variations are fairly well documented in the literature [9,10,17,23].

**Figure 1:**
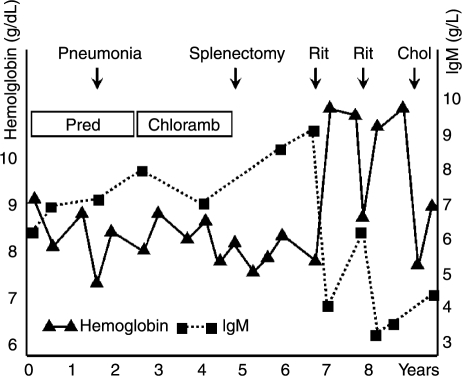
Example of clinical course in primary CAD. Retrospective data from almost ten year follow-up of a female patient, now 74-years old. Abbreviations: Chloramb, chlorambucil; Chol, cholecystitis; Pred, prednisolone; Rit, rituximab.

According to review articles, anemia in CAD is variable and usually not severe [9,17]. However, this is definitively not always the case. Five of 16 patients described in an early report had minimum hemoglobin (Hb) levels below 7.0 g/dl and one below 5.0 g/dl [10]. In a series of 86 patients, we found a median Hb level of 8.9 g/dl, and one-third of the patients had Hb levels at presentation ranging from 4.5 to 8.0 g/dl. Approximately, 50% of the patients were considered transfusion dependent at some time during the course of the disease [3]. Paradoxically, hemolysis is enhanced during febrile illnesses in about two-thirds of the patients [3,4,24,25]. We found no overall change over time in Hb levels and parameters of hemolysis. Hb levels decreased, however, by as much as 7.7 g/dl in individual patients during a median observation time of five years and increased by as much as 5.8 g/dl in others [3]. Thus, CAD tends to be a non-progressive disease in most patients, although fluctuations in the clinical manifestations are prevalent (Figure 1) and it should be emphasized that there are considerable individual variations. The figures clearly document that CAD is not an “indolent” disease in terms of major clinical symptoms and quality of life.

## Immune biology

In the great majority of CAD patients, CA are specific for the I antigen, an erythrocyte surface carbohydrate macromolecule [26,27]. Anti-Pr and anti-P specificities have also been described [27,28]. The concept of CA should not be confused with that of cryoglobulin, although obvious similarities do exist between primary CAD and cryoglobulinemia type I and II [29]. Immunoglobulins have occasionally been described that possess both CA and cryoglobulin properties [30,31].

**Figure 2:**
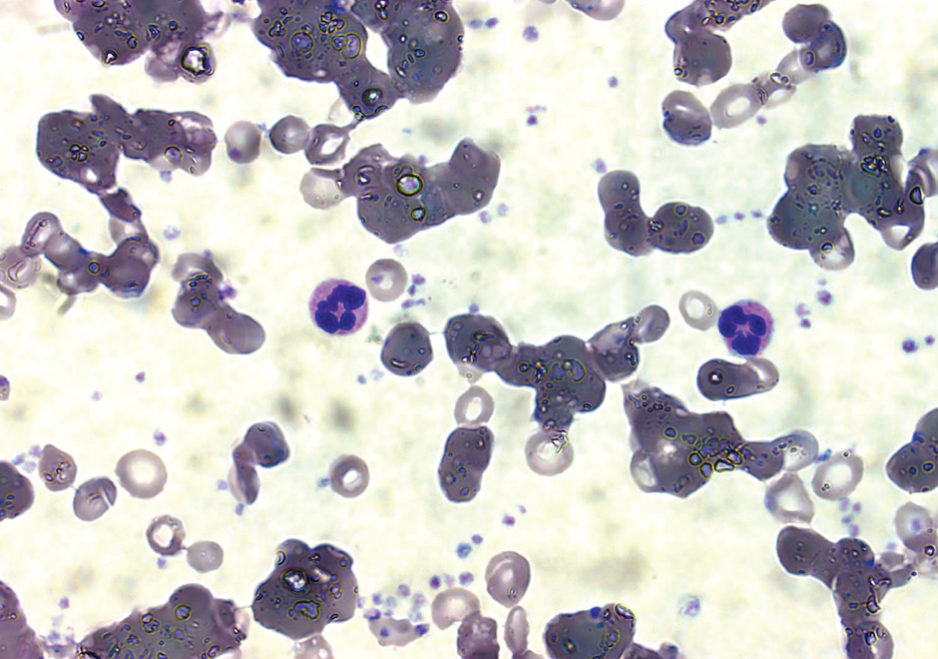
Blood smear from patient with primary CAD. Most erythrocytes are agglutinated in variably large clumps. Giemsa, oil immersion, objective × 100.

**Figure 3:**
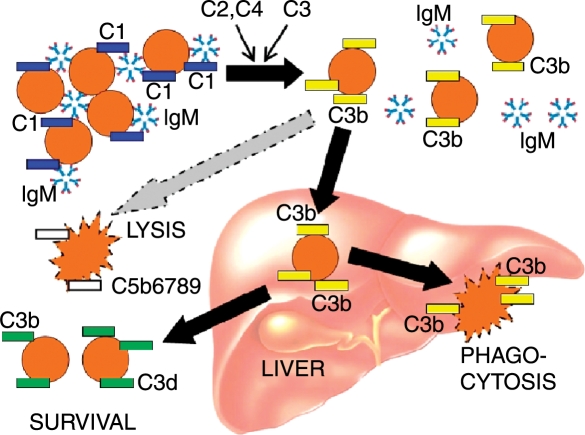
During passage through acral blood vessels, cooling allows IgM cold agglutinin to bind to erythrocytes, causing agglutination and binding of complement C1 complex. C1 esterase activates C4 and C2, generating C3 convertase which binds and splits C3, leading to deposition of C3b on the erythrocyte surface. Upon subsequent warming, IgM removes from the cell surface and the agglutinated cells are detached from each other, while C3b remains bound. C3b may in turn activate C5, leading to the formation of the membrane attack complex and intra vascular cell lysis. Most destruction of C3b-coated erythrocytes, however, is mediated by reticulo-endothelial cells in the liver [15,16,32]. Intrahepatic conversion of C3b is responsible for the deposition of C3d on the surviving erythrocytes which are released into the systemic circulation.

The mechanisms of red-cell agglutination and subsequent destruction have been elucidated in detail [13,15,16,32]. Cooling of blood during passage through acral parts of the body allows CA to bind to erythrocytes and precipitate agglutination (Figure 2). The antigen–antibody complex induces C binding and activation via the classical pathway as shown in Figure 3. Thus, C1 esterase activates C4 and C2, generating C3 convertase which leads to the formation of C3b. Upon subsequent warming to 37°C when the blood returns to the central parts of the body, CA detaches from the cell surface allowing the agglutinated erythrocytes to separate from each other, while C3b remains bound. Some C3b-coated erythrocytes are sequestered and destroyed by C3-receptor bearing reticulo-endothelial cells, mainly in the liver. On the surface of the surviving erythrocytes, C3b is cleaved into C3c and C3d, leaving large numbers of C3d macromolecules on the cell surface. C activation may proceed beyond this step, resulting in C5 activation by C3b and formation of the membrane attack complex C5-9 with intravascular cell lysis. Most evidence suggest, however, that the major mechanism of hemolysis in stable patients is the hepatic sequestration of C3b-coated erythrocytes [5,13,15,16,32].

The thermal amplitude, defined as the highest temperature at which the antibody binds the antigen, appears to be more important than the titer with respect to the pathogenicity of CA [12,13,33]. The CA found in some healthy individuals are usually present in low titers, and titers in excess of 256 are very uncommon in this group [27,34]. Further more, the thermal amplitude of cold-reactive auto antibodies in healthy subjects does not exceed 15–20°C and, therefore, they are of no clinical significance [27].

Christenson [35] and co-workers found in 1957 that CA may sometimes be seen as an abnormal peak in the γ-region by electrophoretic separation of serum proteins on cellulose columns. Fudenberg and Kunkel showed that these antibodies usually have a high (19 S or 1000 kDa) molecular weight [36]. Later, Harboe and co-authors further characterized the CA in CAD as monoclonal IgMκ [37–39].In a recent study of sera from 172 patients with monoclonal IgM associated with a variety of clinical disorders, CA were identified in 10 sera (8.5%) [40]. Both, pentameric and significant levels of hexameric IgM have been detected in samples of purified CA from CAD patients [41]. Absence of J chains seems to enhance the formation of hexameric IgM and has been interpreted as a deleterious feature of IgM-mediated disorders, resulting in a higher ability to activate the C cascade and there by in a higher lytic efficiency of IgM [41,42].

In our population-based descriptive study of primary CAD, a monoclonal band was detected by electrophoresis and immunofixation in sera from 79 (94%) of 84 patients with available data [3]. The monoclonal immunoglobulin was of the IgM class in 71 patients (90%), IgA and IgG in three patients (3.5%) each, while two patients (2.5%) had clonal bands of both IgG and IgM. The light chain restriction was κ in 74 patients (94%), λ in two (2.5%) and unknown in three (3.5%). Since CA detach from the erythrocytes when the blood returns to the body core, specific direct antiglobulin test (DAT) is usually negative when performed with anti-IgM. DAT is positive for C3d by definition (Figure 3) [3,13,15,16]. In our retrospective study [3], specific DAT for IgG was negative in 64 patients (79% of those with available data), while erythrocyte-bound IgG was detected in the remaining 17 (21%). Five patients with monoclonal IgG or biclonal IgM and IgG in serum all displayed IgG on the erythrocyte surface. It has not been established whether this cell-bound IgG is a polyclonal reactive antibody or monoclonal CA of the IgG type.

During maturation, each B-lymphocyte constructs its specific immunoglobulin heavy chain by assembly of coding sequences from the variable (V_H_), diversity (D), and joining (J_H_) gene segments. The diversity created by this recombination process is further increased by enzymatic modification at the cut ends of the gene segments, followed by the event of somatic hypermutation, typically occurring in the hypervariable segments of V_H_ genes. Pascual, Thorpe, Stevenson and others have shown that anti-I CA found in serum samples from patients with primary CAD are preferentially encoded by the V_H_4-34 gene segment, formerly termed V_H_4.21 [43,44]. This gene segment appears to be over represented among the coding unit repertoire, although it accounts for a very small fraction of normal circulating immunoglobulins [43,45]. We assessed the frequency of V_H_4-34 gene expression by testing sera from 11 CAD patients with hemagglutination inhibition assay using the rat monoclonal anti-idiotypic antibody 9G4, which is specific for V_H_4-34 encoded protein. All patient sera were confirmed to be idiotope positive [4]. In contrast, “naturally” occurring CA in healthy individuals, as well as CA artificially induced by Rhesus (D) immunization, are often derived from V_H_ gene segments other than V_H_4-34 [45,46].

## “Paradoxical” exacerbation during febrile illnesses

Reduced C factor levels in CAD were described early by Jonsen [47] and others. In 1998, Ulvestad reported on a patient who experienced that during the course of the disease, the initial cold-induced exacerbations were gradually substituted for “paradoxically” enhanced hemolytic anemia during febrile episodes [24]. The C4 levels decreased steadily and eventually became undetectable, and the *in vitro* hemolytic activity of serum (CH50) declined to zero. In a subsequent study, we assessed C protein levels in 15 CAD patients and found reduced levels of C3 in nine and C4 in 11 patients, six of whom had low CH50 [4]. Based on the records, exacerbation of hemolysis during acute phase reaction had occurred in five patients. In our population-based retrospective study, 64% of CAD patients (50 of 68 patients with available data on such deteriorations) reported exacerbation of hemolytic anemia during febrile illnesses [3].

In order to further investigate these phenomena, we under took a longitudinal, prospective, 12 month follow-up study of one single patient with “paradoxical” exacerbations of hemolysis [25]. In the absence of any acute events, low C3 and undetectable C4 levels were confirmed. We observed a non-functional classical C pathway and a normal alternative pathway. Exacerbation of hemolytic anemia occurred during pneumonia and once more following a hip fracture with subsequent surgery, and was paralleled by increased CRP levels. During each acute event the serum IgM levels declined temporarily, and after the hip fracture we recorded increased C3 levels, detectable C4, significantly increased levels of the pro-inflammatory cytokines interleukin-6, tumor necrosis factor-α and interferon-γ, and slightly increased interleukin-1β [25]. The most plausible explanation for these observations is that a majority of CAD patients have low levels of C3 and especially C4 during steady state due to a continuous consumption. C factor levels, in particular low C4 levels, are rate-limiting for hemolysis. During acute phase reactions, C3 and C4 levels increase due to an enhanced production, resulting in exacerbation of hemolysis.

The findings of C consumption and depletion may have clinical implications. First, administration of C-containing plasma products should probably be avoided. Second, these data explain why a majority of patients with CAD have exacerbations during conditions associated with acute phase reaction. Third, a non-functional classical C pathway may affect the therapeutic potential of monoclonal antibodies in CAD [48–50].

## Clonal B-lymphocytes in primary CAD

Pathogenic B-lymphocyte clones in CAD have been suspected or postulated for decades, based on the findings of monoclonal IgMκ CA in most, if not all patients [3,10,31,37–39]. More recently, it has been possible to verify such cell clones directly. Flow cytometric investigations by Silberstein and co-workers disclosed B-cell clones in at least some patients [51]. In 1995, we reported the findings of lymphoplasmacytic lymphoma in the bone marrow of three consecutive patients otherwise classified as having primary CAD [52]. In a subsequent study by our group, patients with no clinical or radiological evidence of an underlying lymphoma were examined by flow cytometric immunophenotyping of bone-marrow aspirates as well as morphological and immunohistochemical assessment of trephine biopsies [31]. A lymphoproliferative bone-marrow disorder characterized by clonal CD19^+^CD20^+^κ^+^ lymphocytes was detected in 10 of 11 patients.

**Figure 4:**
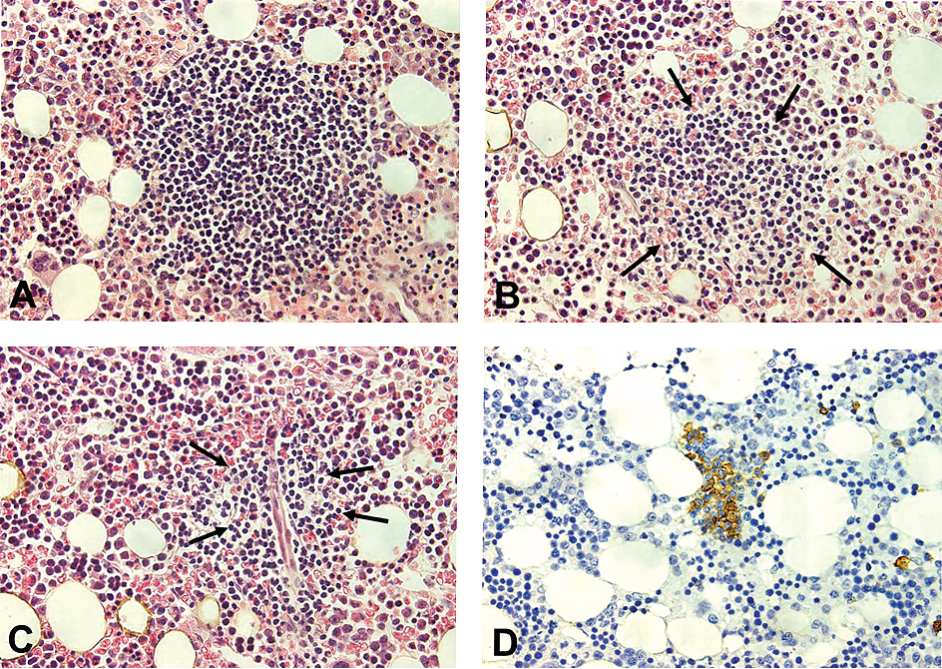
Histopathological appearances in bone marrow trephine biopsy from a patient with primary CAD. Lymphoid infiltrates may be of variable size; large (A), medium-sized (B), or often small and poorly outlined (C) which renders them barely detectable within areas of hyperplastic erythropoiesis unless immunohistological staining is applied (D). A–C, HE-stain; D, Anti-CD20, horseradish peroxidase/diaminobenzidine. All photomicrographs are taken at identical magnification (× 40 objective) to enable comparison of individual infiltrates.

In a recent retrospective study, the medical records of 86 patients otherwise classified as having primary CAD were re-examined with regard to the presence of a clonal lymphoproliferative bone-marrow disorder [3]. Monoclonal CD20^+^κ^+^ lymphocytes were found in the bone marrow of most patients in whom a flow cytometric assessment had been performed. Based on previously published data [31,53], a κ/λ ratio > 3.5 by flow cytometry was considered strongly indicative of a clonal lymphoproliferative B-cell disorder. The median κ/λ ratio was 7.8 (range 0.9–186), and a ratio higher than 3.5 was found in 36 (90%) of 40 patients with available data [3]. Data on bone-marrow histology are shown in Table I. Morphologic and immunohistochemical signs of non-Hodgkin's B-cell lymphoma were found in 50 (76%) of 66 patients with available information (Figure 4). Applying the WHO classification [54], 33 patients had lymphoplasmacytic lymphoma (50% of patients with available histology data and 66% of those with a demonstrable clonal lymphoproliferative bone marrow disorder).

**Table I:** Bone marrow histology in patients with primary CAD.

	*n*	%		
Normal findings or reactive lymphocytosis	7		11	
Irregular lymphoid hyperplasia	9		13	
Non-Hodgkin's B cell lymphoma	50		76	
Lymphoplasmacytic lymphoma		33		50
Marginal zone lymphoma		5		8
Small lymphocytic lymphoma/chronic lymphocytic leukemia		4		6
Clonal lymphocytosis/other small B cell lymphoma		8		12
Total	66		100	

According to recent criteria, Waldenström's macro-globulinemia (WM) is defined as lymphoplasmacytic lymphoma of the bone marrow combined with monoclonal IgM at any serum concentration [55]. When these criteria were applied, 50% of CAD patients with available immunoglobulin and histology data met the diagnostic criteria for both primary CAD and WM [3]. On the other hand, we have observed an occasional CAD patient with monoclonal IgMκ for more than 21 years without any demonstrable clonal B-cell population as repeatedly assessed by flow cytometry and immunohistochemistry. Transformation to diffuse large B-cell lymphoma appears to be a rare event, occurring in 3–4% of patients with primary CAD after a disease duration of 10 years [3].

Cytogenetic features have been difficult to assess, probably because the cell clones usually are small and the neoplastic cells are indolent and hard to make proliferate in cultures. Trisomy 3q and translocation 8;22, respectively, have been reported in single cases [56,57].

CAD patients diagnosed by us and others to have a low-grade lymphoproliferative bone marrow disorder undoubtedly represent the same majority that used to be classified as having primary CAD [9,27,31,48]. Except in the uncommon event of transformation, these clonal lymphoproliferative disorders seldom, if ever, show features of clinically overt lymphoma even after decades [3]. Furthermore, most of the rare patients traditionally classified as having secondary CAD suffer from a readily demonstrable lymphoma, often of an aggressive type, that may be associated with IgMλ as well as IgMκ CA [58,59]. Therefore, we still think it is appropriate to apply the term primary CAD in patients not showing the classical features of the secondary type.

## Diagnosis

Based on the characteristics discussed in the preceding paragraphs and available literature [1,3,5,10,17,27], the criteria shown in Table II should be used to define primary CAD. The demonstration of a monoclonal serum immunoglobulin and a clonal, lymphoproliferative bone-marrow disorder should not be regarded as an absolute prerequisite for diagnosis, since the cell clones may be too small to manifest themselves by histopathologic findings or be detected by flow cytometry, electrophoresis and immunofixation. Such verification of clonality depends to a large extent on sensitivity and, in particular with respect to the electrophoretic findings, on optimal preparation and examination of specimens.

**Table II:** Diagnosis of primary CAD.

		Comments and precautions
Criteria	Chronic hemolysis	
	Cold agglutinin titer ≧ 64 at 4°C	
	Typical DAT findings:	Specific DAT for IgG is usually, but not always, negative
	Polyspesific DAT positive	
	Specific DAT positive for C3d	
	No malignant disease by clinical and radiological assessment	
Procedures: blood and serum	Hemoglobin level and blood cell counts	
	Routine assessment for hemolysis	
	DAT. Specific DAT for C3d and IgG	
	Cold agglutinin (CA) titer at 4°C	Blood specimens for CA and immunoglobulin analyses must be kept at 37°C from sampling until serum has been removed from the clot
	Complement assessments (C3, C4 and CH50)	
	Electrophoresis with immunofixation	
	Quantification of IgM, IgG and IgA	Immunofixation should be performed even if no monoclonal band is visible on electrophoresis
Procedures: bone marrow	Trephine biopsy (including immunohistochemistry)	Morphology and immunohisto-chemistry of trephine biopsies should be assessed by an experienced hemopathologist
	Flow cytometry of aspirate	
Radiology	Chest X-ray	
	Abdominal ultrasonography	

Table II lists the diagnostic examinations that should be performed. Problems in measurement of blood cell counts may sometimes been countered due to agglutination, but pre-warming of the EDTA-blood samples when necessary will eliminate such difficulties. For serum immunoglobulin analyses, including cold agglutinin titration, electrophoresis, immunofixation and quantification of immunoglobulin classes, it is essential to keep blood specimens at 37°C from sampling until serum has been removed from the clot. Assessment of thermal amplitude may be informative, but is hardly needed for diagnostic or therapeutic decisions. Bone-marrow examination by flow cytometry of aspirate and careful assessment of a trephine biopsy sample should always be performed.

## Management of primary CAD

According to literature, counseling on cold avoidance should be the mainstay in management of primary CAD [17,19,60]. In 63 (73%) of 86 patients reported by us, however, the physician and/or the patient had not perceived such measures as sufficient [3]. Corticosteroids and alkylating agents are usually ineffective [3,19,20]. Improvement following interferon-a single agent therapy has been reported in a small retrospective series, but in another series none of the patients responded [61,62]. Furthermore, no response to cladribine monotherapy was observed in a small, prospective study, but the doses of cladribine applied in this trial were low [63]. The potential of splenectomy has not been studied systematically, but theoretical considerations and clinical experience strongly discourage its use as a therapeutic procedure [3,15,16,19,32].

The recognition of primary CAD as a clonal lymphoproliferative CD20^+^B-cell disorder and the success of treatment with the monoclonal anti-CD20 antibody rituximabin CD20^+^ non-Hodgkin's lymphoma [64,65] made us and other investigators hypothesize that rituximab therapy might also be effective in CAD. The adverse effects of rituximab are different from those of most cytotoxic drugs and less severe [64,65], and the B-lymphocyte elimination is not cell cycle dependent [66]. One small and two somewhat larger phase 2 trials [48,67,68] have been published in addition to a number of case reports [69]. In the first 16 case reports published, all patients improved after rituximab therapy, and a high proportion of the responses were classified as complete [70,71]. The explanation for such a high response rate is probably that response rates estimated from case reports are likely to be strongly influenced by publication bias, lack of strict disease definitions, and heterogeneous or lacking response criteria.

We reported on 37 courses of rituximab single agent therapy administered to 27 patients with primary CAD in a prospective, uncontrolled trial [48]. Each eligible patient received a course of rituximab at a dose of 375 mg/m^2^ on day 1, 8, 15 and 22. Re-treatment was permitted in patients who responded and subsequently relapsed. The response criteria are summarized in Table III. Fourteen of 27 patients responded to their first course of rituximab, and six of ten relapsed patients responded to re-treatment. In both groups combined, responses were achieved after 20 of 37 courses, resulting in an overall response rate of 54%. We observed one complete and 19 partial responses. Responders achieved a median increase in Hb levels of 4.0 g/dl and a median decrease in IgM levels by 54%. Clinical and laboratory data indicated a benefit even in some patients classified as non-responders. Median time to response was 1.5 months (range, 0.5–4.0) and median observed response duration was 11 months (range, 2–42). No serious adverse events occurred. The results of a similar trial in 20 patients by Schöllkopf and co-workers fit in very well with our findings, although they reported a shorter response duration [68]. Some minor discrepancies between the results of the two studies may be explained by slightly different inclusion and response criteria.

**Table III:** Response criteria used in therapeutic trials.

Complete response	Absence of anemia
	No signs of hemolysis
	Disappearance of clinical symptoms of CAD
	Undetectable monoclonal serum protein
	No signs of clonal lymphoproliferation as assessed by bone marrow histology, immunohistochemistry and flow cytometry
Partial response	A stable increase in hemoglobin levels by at least 2.0 g/dl or to the normal range
	A reduction of serum IgM concentrations by at least 50% of the initial level or to the normal range
	Improvement of clinical symptoms
	Transfusion independence
No response	Failure to achieve complete or partial response
In order to qualify for any given response level, all criteria have to be fulfilled	

## Possible directions for future research

The benefit achieved by rituximab single agent therapy in CAD is limited by a 45–50% failure rate and relatively short response duration. Further studies are warranted, therefore, in order to explain the variable effect of rituximab therapy, identify possible predictors, and improve on response rates and response duration.

Even when a CD20^+^κ^+^ lymphocyte clone can merely be detected and monoclonal IgM is present at low levels, patients may have a clinically severe disease with a high CA titer or CA with high thermal amplitude [4,31]. Small B-cell clones that produce deleterious proteins are well known, and these conditions are very often difficult to treat effectively [72,73]. Thus, an explanation for the difficulties in achieving remissions may be that in most cases, small cell clones produce biologically highly active antibodies that must be nearly eradicated in order to achieve clinical improvement. On the other hand, rituximab can induce good partial remissions even in patients who achieve only a modest decrease in monoclonal IgM by about 50% [48]. This may indicate that reduction of the lymphocyte clone and the concentration of the auto-antibody may not be the only pathway of therapeutic effect. In WM, the monoclonal B-cell population can induce expansion of circulating, polyclonal B-lymphocytes [74]. To our knowledge, no studies have been done to explore the possible role of this phenomenon in CAD or any implications for therapy.

Rituximab has been shown to kill CD20^+^ cells by at least three mechanisms; C-dependent cytotoxicity (CDC), antibody-directed cellular cytotoxicity (ADCC), and induction of apoptosis by direct intracellular signaling [66]. Some *in vitro* and *in vivo* data indicate that CDC is an essential mechanism of action and, therefore, the reduced availability of C proteins in many patients with CAD may turnout to be of clinical importance [49,50]. In our prospective trial, however, we found no association between C3 or C4 levels and response to rituximab therapy [48]. The administration of interferon-a may raise serum C4 levels [75] and up-regulate CD20 expression on the surface of B-cells [76,77]. In our rituximab study, we intended to evaluate whether combining rituximab and interferon-α could improve on efficacy [48]. Patient or physician preferences, however, resulted in only five patients receiving the combination, and it was impossible to put forward any firm statements on the efficacy of combining rituximab with interferon-α.

Elimination of CD20^+^ lymphocytes by anti-CD20 induced ADCC requires binding of the Fc-domain of the CD20-bound antibody to the Fc-receptor of effector cells [66]. Polymorphism in the IgG Fcγ receptor IIIa (Fcγ-RIIIa) gene has been proposed to influence the depletion of B-lymphocytes by rituximab [78,79]. Although the possible consequences of such genetic variations remain to be confirmed in CAD, clinical studies have suggested that Fcγ-RIIIa polymorphism may explain the variability in the response to rituximab therapy in WM [80].

Purine analogues have shown a remarkable efficacy in low-grade lymphoproliferative diseases, including WM [81,82]. Although purine analogues do not seem promising in CAD when administered as monotherapy [63], remission has been reported in two single cases after the administration of cladribine and fludarabine, respectively [3,83]. In a small, prospective study, cladribine was shown to reduce the number of clonal cells, although not resulting in any significant clinical improvement [63]. A synergistic effect of fludarabine and rituximab have been shown in a follicular lymphoma B-cell line resistant to the cytotoxic activity of either drug alone, probably mediated through a down-modulation of membrane CD55 [84]. In WM, purine analogue and rituximab combination therapy has resulted in higher response rates and more prolonged remissions as compared to purine analogue single agent therapy [85]. Fludarabine may induce AIHA, but this adverse event seems to occur mainly in patients with chronic lymphocytic leukemia, and recent observations may indicate that the addition of rituximab will reduce the risk [86].

We are now running a phase 2 study on the safety and efficacy of rituximab and fludarabine combination therapy in primary CAD [87], still using the response criteria listed in Table III. By February 2007, response evaluation was possible in the first nine patients, median age 72 years (range, 59–85). Six had previously received rituximab single-agent therapy, resulting in one complete response and one partial response, while four had been non-responders. Following combination therapy, four patients achieved a complete response, four achieved a partial response and one did not respond. Hematologic toxicity was observed in four patients (grade 2, 3 and 4, respectively) and infection grade 2, nausea and dermatitis in one each. Thus, rituximab and fludarabine combination therapy seems feasible even in elderly patients with CAD. Response rates are promising and suggestive of a higher efficacy, but superiority over rituximab single-agent therapy remains to be proven in an extended study.

Since the hemolytic activity of CA is C dependent, one might consider direct C modifying agents as possible therapeutic options. Infusion of the humanized, monoclonal anti-C5 antibody eculizumab has recently been documented as a powerful therapeutic measure in paroxysmal nocturnal hemoglobinuria [88]. No reports have been published on its use in CAD. Based on the mechanisms of CA mediated C activation and hemolysis discussed in the previous paragraphs, however, one should theoretically not expect a pronounced effect in stable CAD patients. Prospective trials may still be justified in refractory patients with severe hemolysis or acute exacerbations.
